# Formic acid enhances whole-plant mulberry silage fermentation by boosting lactic acid production and inhibiting harmful bacteria

**DOI:** 10.3389/fmicb.2024.1399907

**Published:** 2024-06-10

**Authors:** Lihong Hao, Fugui Jiang, Yanping Wang, Huaizhong Wang, Hongmei Hu, Wei You, Xin Hu, Haijian Cheng, Cheng Wang, Enliang Song

**Affiliations:** ^1^Shandong Key Laboratory of Animal Disease Control and Breeding, Institute of Animal Science and Veterinary Medicine, Shandong Academy of Agricultural Sciences, Jinan, China; ^2^Key Laboratory of Livestock and Poultry Multi-Omics of MARA, Jinan, China; ^3^College of Life Sciences, Shandong Normal University, Jinan, China

**Keywords:** mulberry, silage, glucose, formic acid, lactic acid bacteria, salts, bacterial community

## Abstract

Mulberry has also been regarded as a valuable source of forage for ruminants. This study was developed to investigate the impact of four additives and combinations thereof on fermentation quality and bacterial communities associated with whole-plant mulberry silage. Control fresh material (FM) was left untreated, while other groups were treated with glucose (G, 20 g/kg FM), a mixture of *Lactobacillus plantarum* and *L. buchneri* (L, 10^6^ CFU/g FM), formic acid (A, 5 mL/kg FM), salts including sodium benzoate and potassium sorbate (S, 1.5 g/kg FM), a combination of G and L (GL), a combination of G and A (GA), or a combination of G and S (GS), followed by ensiling for 90 days. Dry matter content in the A, S, GA, and GS groups was elevated relative to the other groups (*p* < 0.01). Relative to the C group, all additives and combinations thereof were associated with reductions in pH and NH_3_-N content (*p* < 0.01). The A groups exhibited the lowest pH and NH_3_-N content at 4.23 and 3.27 g/kg DM, respectively (*p* < 0.01), whereas the C groups demonstrated the highest values at 4.43 and 4.44 g/kg DM, respectively (*p* < 0.01). The highest levels of lactic acid were observed in the GA and A groups (70.99 and 69.14 g/kg DM, respectively; *p* < 0.01), followed by the GL, L, and GS groups (66.88, 64.17 and 63.68 g/kg DM, respectively), with all of these values being higher than those for the C group (53.27 g/kg DM; *p* < 0.01). *Lactobacillus* were the predominant bacteria associated with each of these samples, but the overall composition of the bacterial community was significantly impacted by different additives. For example, *Lactobacillus* levels were higher in the G, A, and GA groups (*p* < 0.01), while those of *Weissella* levels were raised in the L, GL, and GS groups (*p* < 0.01), *Pediococcus* levels were higher in the A and GA groups (*p* < 0.01), *Enterococcus* levels were higher in the G and S groups (*p* < 0.01), and *Lactococcus* levels were raised in the S group (*p* < 0.01). Relative to the C group, a reduction in the levels of undesirable *Enterobacter* was evident in all groups treated with additives (*p* < 0.01), with the greatest reductions being evident in the A, S, GA, and GS groups. The additives utilized in this study can thus improve the quality of whole-plant mulberry silage to varying extents through the modification of the associated bacterial community, with A and GA addition achieving the most efficient reductions in pH together with increases in lactic acid content and the suppression of undesirable bacterial growth.

## 1 Introduction

Mulberry (*Morus alba L.*) is a woody perennial plant belonging to the Moraceae family that is highly adaptable and widely distributed throughout China. Parts of the mulberry plant, particularly the leaves, are frequently used in the practices of traditional Chinese medicine as they are rich in phenols, polysaccharides, alkaloids, and other phytochemicals such that they possess an array of anti-inflammatory, antioxidant, antidiabetic, antihelmintic, and antimicrobial properties (He et al., 2018; Zhang et al., 2018; Shan et al., 2022). Mulberry has also been regarded as a valuable source of forage for ruminants, as it exhibits high protein content, contains high levels of vitamins and minerals, and presents with a high biomass yield (Thaipitakwong et al., 2018). The high moisture content in fresh mulberry plants, however, makes them difficult to preserve, particularly during the rainy season in Southern China. While silage represents an effective approach to conserving fresh material, the fermentation process is complex and subject to numerous influencing factors, resulting in variability in silage quality that may require the incorporation of additives to properly manage the fermentation process (Yitbarek and Tamir, 2014). The generation of mulberry silage of superior quality remains difficult due to its low contents of water-soluble carbohydrates (WSC) and high buffering capacity (Wang et al., 2019a). There is thus a pressing need for the use of rational strategies to select appropriate additives that can be applied during ensiling in order to produce higher quality silage.

A variety of silage additives can be used to enhance fermentation, increase silage nutritional value, minimize losses, protect against aerobic deterioration, and thereby achieve improved aerobic stability (Yitbarek and Tamir, 2014). These additives can be broadly classified into stimulants and inhibitors of fermentation, inhibitors of aerobic deterioration, absorbents, and nutrients (McDonald et al., 1991). Glucose can serve as a fermentation stimulant by increasing the amount of fermentable sugar substrates accessible to lactic acid bacteria (LAB) during ensiling, supporting a rapid decrease in pH and enhanced lactic fermentation through the inhibition of proteolytic and butyric fermentation (Trabi et al., 2017). Li et al. (2014) found that glucose was able to improve king grass ensiling fermentation by facilitating the rapid accumulation of lactic acid during the early period of ensiling. LAB present in silage consist of homofermentative and heterofermentative LAB (Muck et al., 2018). Homofermentative LAB can rapidly and robustly reduce pH levels during ensiling through their conversion of WSC into lactic acid (Zhang et al., 2019). *Lactobacillus buchneri* is the dominant obligate heterofermentative LAB species used as a silage additive, functioning during the later stages of ensiling by the gradual conversion of lactic acid into acetic acid and 1,2-propanediol, thereby leading to enhanced aerobic stability (Muck et al., 2018). In addition, formic acid has been utilized as a fermentation inhibitor through its ability to achieve a rapid decline in pH through direct acidification, suppressing the growth of undesirable spoilage-associated microbes including enterobacteria and aerobic bacteria while allowing an optimal environment for more rapid LAB growth and improved silage preservation (Yitbarek and Tamir, 2014). Jiang et al. (2020) found that treating of whole-plant corn silage with formic acid, acetic acid, and potassium sorbate at a 7:1:2 ratio (6 L/t) was associated with increased lactic acid levels and a reduction in the levels of undesirable microbes including *Klebsiella*, *Paenibacillus*, and *Enterobacter*. Salts including sodium benzoate and potassium sorbate can effectively reduce the growth of yeast, molds, and spoilage-related bacteria in silage, thereby leading to increased aerobic stability. Knický and Spörndly (2009) found that when ensiling grasses with low dry matter (DM) content, sodium benzoate and potassium sorbate treatment were associated with higher lactic acid levels, together with lower levels of butyric acid and NH_3_-N relative to untreated silage. This suggests that adding glucose may provide a means of overcoming the lack of WSC content in mulberry plants, while the application of LAB, organic acids, salts, or combinations thereof may help further improve fermentation quality for whole-plant mulberry silage.

Improvements in silage quality through the use of any additive types are ultimately attributable to effects on the activity of beneficial or undesirable microbes (Kung et al., 2018; Liu et al., 2019). The development of high-throughput sequencing has fueled a growing number of studies exploring how various additives impact microbial communities in stylo (He et al., 2021), amaranth (Zhao et al., 2022), paper mulberry (Cheng et al., 2022), and native grass (Li et al., 2022). As far as we know, previous studies mainly used mulberry leaves as raw materials to prepare silage, focusing on the effects of additives on chemical composition, fermentation quality and part of microorganisms, rather than the whole plant (He et al., 2019; Wang et al., 2019a; Dong et al., 2020; Wang C et al., 2020; Cui et al., 2022). Little remains known, however, on the influence of different types of additive on fermentation and variations in microbial communities associated with whole-plant mulberry silages. As such, the present study was conceived with the goal of assessing how glucose, LAB, organic acids, salts and mixtures thereof impact fermentation quality and bacterial communities during mulberry ensiling through a high-throughput sequencing approach. The findings provided by this study can serve as a reference for addressing feed shortages, enhancing the quality of forage feed, and improving the economic management of ruminant husbandry.

## 2 Materials and methods

### 2.1 Plant materials and silage treatment

Whole mulberry plants were manually collected from a field in Changle County, Weifang City (118°83′E, 36°69′N), Shandong, China on September 25th, 2023. Harvested materials were manually chopped using a chopper (FS-690, Zili, China) to produce 2 cm segments. Mulberry material chemical composition before ensiling is shown in Table 1. The lactic acid bacteria (LAB), glucose, formic acid, and salts were employed as additives during ensiling. LAB treatment consisted of a 9:1 combination of *Lactobacillus plantarum* and *Lactobacillus buchneri*, which was used to inoculate samples at 10^6^ colony-forming units (CFU) per gram of fresh material (FM). Glucose and formic acid were applied to the silage at 2 and 0.5% of FM, respectively. Salts consisted of a 2:1 (m/m) mix of potassium sorbate and sodium benzoate that was applied to silage at 1.5 g/kg of FM. The chopped mulberry material was mixed thoroughly, and separated into eight equal-sized parts for the following treatment conditions: no additive (C), glucose (G), LAB (L), formic acid (A), salts (S), G + L (GL), G + A (GA), G + S (GS). The mixed forage (1,000 g) was packed into plastic bags (20 × 30 cm; Deli Group, China), using vaccum sealing (Deli 14,886, Deli Group) for air removal. Four replicates per treatment were established for 32 total silage samples (8 treatments × 4 replicates), and these samples were stored in the dark for 90 days of ensiling at ambient temperature (21–25°C). The study period was from September 2023 to February 2024.

**Table 1:** Chemical composition of fresh whole-plant mulberry (Mean ± SD, *n* = 4).

Items	Fresh whole-plant mulberry
DM (% FM)	40.69 ± 0.10
CP (% DM)	14.48 ± 0.55
NDF (% DM)	41.26 ± 1.62
ADF (% DM)	18.96 ± 0.67
ADL (% DM)	6.89 ± 0.40
Ash (% DM)	10.70 ± 0.06
EE (% DM)	1.66 ± 0.15
WSC (% DM)	4.96 ± 0.10
Lactic acid bacteria (log cfu/g FM)	3.72 ± 0.24
Yeast (log cfu/g FM)	5.33 ± 0.47

FM, fresh; DM; dry matter; CP, crude protein; NDF, neutral detergent fiber; ADF, acid detergent fiber; ADL, acid detergent lignin; EE, ether extract; WSC, water soluble carbohydrate.

### 2.2 Chemical composition and microbial population analyses

A 1-mm screen was used to grind samples following incubation for 48 h at 65°C for chemical analysis. Dry matter (DM) contents were assessed by dying samples to a constant weight for 3 h at 105°C. WSC was analyzed as detailed previously by Murphy (1958). Acid detergent fiber (ADF) and neutral detergent fiber (NDF) were determined as in a prior report (Van Soest et al., 1991). Crude protein (CP), acid detergent lignin (ADL), ether extract (EE), and ash were analyzed as per protocols published by the Association of Official Analytical Chemists (AOAC, 1990).

Microbial population analyses were performed by mixing 20 g of fresh sample with sterile saline solution (0.85% NaCl), followed by serial dilution from 10^−1^ to 10^−7^. LAB were then counted using the plate count method on de Man, Rogosa, and Sharpe (MRS) agar after anaerobic incubation for 48 h at 37°C. Yeast were counted after aerobic incubation for 48 h at 30°C on potato dextrose agar (PDA). Colony counts were reported as the number of viable microbes, with these values being transformed into log10 cfu/g FM.

### 2.3 Analyses of fermentation characteristics

Silage samples (20 g) were combined with sterile water (180 mL), homogenized for 60 s using a blender, and filtered by passing the solution through four cheesecloth layers. The resultant filtrate was then immediately tested to determine its pH using a pH meter (HI-9126; Hanna Instruments, United States). Ammoniacal nitrogen (NH_3_-N) content was measured as reported previously by Broderick and Kang (1980). The levels of organic acids (lactic, acetic, propionic, and butyric acids) were measured via PLC using a Shodex RSpak KC-811S-DVB gel C column (Shimadzu, Japan), using a mobile phase consisting of 3 mmol/L HClO_4_ and a 1.0 mL/min flow rate.

### 2.4 Microbiome analyses

#### 2.4.1 DNA extraction and PCR amplification

Samples (50 g) were collected and immediately frozen for future extraction of bacterial DNA performed with the E.Z.N.A.® soil DNA Kit (Omega Bio-tek, GA, United States) as directed. The concentration and purity of the DNA were analyzed with a NanoDrop 2000 UV–vis spectrophotometer (Thermo Scientific, DE, United States) and 1% agarose gel electrophoresis (AGE), respectively.

The V3-V4 region of the 16S rRNA gene was amplified via PCR with the 338F (5’-ACTCCTACGGGAGGCAGCAG-3′) and 806R (5’-GGACTACHVGGGTWTCTAAT-3′) primers using a GeneAmp 9,700 instrument (ABI, United States). Amplified products were separated via 2% AGE and purified using an AxyPrep DNA Gel Extraction Kit (Axygen Biosciences, CA, United States) and quantified using QuantiFluor™-ST (Promega, Madison, WI, United States).

#### 2.4.2 Sequencing analyses

Equimolar amounts of the purified amplicons were pooled. Paired-end sequencing (2 × 300) was performed on an Illumina Miseq PE300 platform (Illumina, CA, United States). Trimmomatic was used to quality filter the resultant paired-end reads, eliminating sequences of low quality (average quality score < 20) or ambiguous bases. Pre-processes reads were merged using FLASH (v1.2.11) with a minimum 10 bp overlap and a 2% mismatch error rate. QIIME v1.8.0 was then used for sequence denoising, with the detection and removal of chimeric sequences. Operational taxonomic units (OTUs) were clustered using UPARSE (v11) with a cutoff level of 97% similarity. OTUs were standardized based on the sample with the lowest sequence number to facilitate further analyses. Taxonomic analyses were conducted using the Silva (SSU138) database to align 16S rRNA sequences with the RDP classifier algorithm (v2.13) at a 70% confidence threshold. Venn diagram analyses were used to detect unique and overlapping OTUs among groups. MOTHUR (v1.30.2) was used for the calculation of alpha diversity indices (Ace, Chao, Shannon, Simpson, and Coverage), with differences among groups being analyzed with the Kruskal-Wallis H test with the Tukey–Kramer *post hoc* test. A weighted unifrac distance-based principal coordinate analysis (PCoA) was performed in QIIME (v1.9.1) to assess beta diversity, while differences among groups were examined using analysis of similarities (ANOSIM) with 999 permutations. Differences in genus-level bacterial abundance among groups were analyzed with Kruskal–Wallis *H*-tests with the Tukey–Kramer *post hoc* test.

#### 2.4.3 Correlation analyses

An RDA approach was used to probe the relationships among fermentation characteristics and samples at the genus level with the “vegan” package in R, utilizing fermentation characteristics as explanatory variables. Prior to modeling, the variance inflation factor (VIF) was used to select explanatory variables, omitting those variables with a VIF > 10. Correlations between microbial genera and fermenation characteristics were assessed through Spearman’s rank correlation analyses, and a heatmap was used to present the resultant correlation matrix.

#### 2.4.4 Statistical analyses

Fermentation characteristics and chemical composition data were analyzed with one-way ANOVAs and Duncan’s multiple range test using the GLM procedure in SAS (V9.1). Results are presented as least squares means. *p* ≤ 0.05 was regarded as significant unless otherwise indicated, while trends were identified using 0.05 < *p* ≤ 0.10.

## 3 Results

### 3.1 Chemical and microbial composition of fresh mulberry

The fresh mulberry samples used for this study had a DM content of 40.69% (Table 1). On the basis of DM, the CP, NDF, ADF, ADL, Ash, EE and WSC of mulberry were 14.48, 41.26, 18.96, 6.89, 10.70, 1.66, and 4.96%, respectively. The respective LAB and yeast counts for these mulberry samples were 3.72 and 5.33 log cfu/g of FM.

### 3.2 Mulberry silage chemical composition

Similar DM levels were observed in the silage from the A, S, GA, and GS groups, seen in increased values relative to the C, G, and L groups (*p* < 0.05, Table 2). The different treatments had no significant impact on CP, NDF, ADF, or ADL levels (*p* > 0.05). The GL group contained the greatest amount of EE (2.36%), followed by the L group (2.03%), GS group (1.54%), and S group (1.39%), in which the values were greater than those in the all the remaining groups (*p* < 0.01). Relative to the C group, the L and S groups exhibited increased Ash contents (*p* < 0.01), while these levels were reduced in the G and GA groups (*p* < 0.01). The highest WSC content was observed in the GA group (2.83%), followed by the G group (1.75%), A group (1.63%), and GS group (1.59%), with these groups exhibiting values higher than those for all other groups (*p* < 0.01).

**Table 2:** Chemical composition of whole-plant mulberry silage.

Items	Treatments								SEM	*p*-value
	C	G	L	A	S	GL	GA	GS		
DM (% FM)	38.93^d^	39.46^cd^	39.39^cd^	40.16^ab^	39.85^bc^	39.54^bcd^	40.16^ab^	40.60^a^	0.11	<0.001
CP (% DM)	15.39	15.50	15.77	15.93	14.74	15.41	15.73	14.70	0.18	0.649
NDF (% DM)	39.72	39.36	39.30	40.06	40.61	39.09	38.99	38.92	0.42	0.983
ADF (% DM)	18.81	17.69	17.57	18.12	17.91	17.54	17.45	17.53	0.17	0.538
ADL (% DM)	6.12	5.55	5.31	5.85	5.68	5.43	5.69	5.60	0.10	0.681
Ash (% DM)	11.31^bc^	10.99^d^	11.56^b^	11.13^cd^	12.07^a^	11.17^cd^	10.91^d^	11.08^cd^	0.07	<0.001
EE (% DM)	0.80^d^	0.76^d^	2.03^b^	0.75^d^	1.39^c^	2.36^a^	0.96^d^	1.54^c^	0.11	<0.001
WSC (% DM)	0.75^c^	1.75^b^	0.88^c^	1.63^b^	0.80^c^	1.03^c^	2.83^a^	1.59^b^	0.12	<0.001

Means with different lowercase letters in the same row (a–d) differ significantly (*p* < 0.05). C, control; G, glucose; L, lactic acid bacteria; A, formic acid; S, salts; GL, glucose + lactic acid bacteria; GA, glucose + formic acid; GS, glucose + salts; SEM, standard error of means; DM, dry matter; CP, crude protein; NDF, neutral detergent fiber; ADF, acid detergent fiber; ADL, acid detergent lignin; EE, ether extract; WSC, water soluble carbohydrate.

### 3.3 Mulberry silage fermentation characteristics

Samples from the C group presented with the highest pH, propionic acid, butyric acid and NH_3_-N levels, together with the lowest level of lactic acid (Table 3). The lowest pH, propionic acid, and butyric acid levels, in contrast, were observed in the A and GA groups, while NH_3_-N content was lowest in the GA group. Significantly higher lactic acid levels were observed in the L, A, GL, GA, and GS groups relative to the C group (*p* < 0.05), although no marked differences were seen among these groups (*p* > 0.05). The lowest acetic acid contents were observed in the GS group, with these levels being similar to those in the C and S groups and below those of other groups (*p* = 0.017). Relative to the control groups, all additive groups presented with lower propionic acid, butyric acid, and NH_3_-N contents (*p* < 0.01).

**Table 3:** Fermentation characteristics of whole-plant mulberry silage.

Items	Treatments								SEM	*p*-value
	C	G	L	A	S	GL	GA	GS		
pH	4.43^a^	4.31^c^	4.28^cd^	4.23^e^	4.35^b^	4.25^de^	4.23^e^	4.30^c^	0.01	<0.001
Lactic acid (g/kg DM)	53.27^d^	60.15^bcd^	64.17^abc^	69.14^a^	58.90^cd^	66.88^ab^	70.99^a^	63.68^abc^	1.23	<0.001
Acetic acid (g/kg DM)	10.81^bcd^	11.47^ab^	11.18^abc^	11.62^a^	10.63^cd^	11.19^abc^	11.23^abc^	10.37^d^	0.10	0.017
Propionic acid (g/kg DM)	0.80^a^	0.73^b^	0.71^b^	0.33^e^	0.38^d^	0.39^d^	0.33^e^	0.49^c^	0.03	<0.001
Butyric acid (g/kg DM)	0.08^a^	0.05^b^	0.05^bc^	0.02^d^	0.04^c^	0.04^bc^	0.02^d^	0.04^bc^	0.00	<0.001
NH_3_-N (g/kg DM)	4.44^a^	3.79^bc^	3.36^cd^	3.38^cd^	3.91^b^	3.30^d^	3.27^d^	3.78^bc^	0.08	<0.001

Means with different lowercase letters in the same row (a–e) differ significantly (*p* < 0.05). C, control; G, glucose; L, lactic acid bacteria; A, formic acid; S, salts; GL, glucose + lactic acid bacteria; GA, glucose + formic acid; GS, glucose + salts; SEM, standard error of means.

### 3.4 Microbial community of mulberry silage

**Figure 1:**
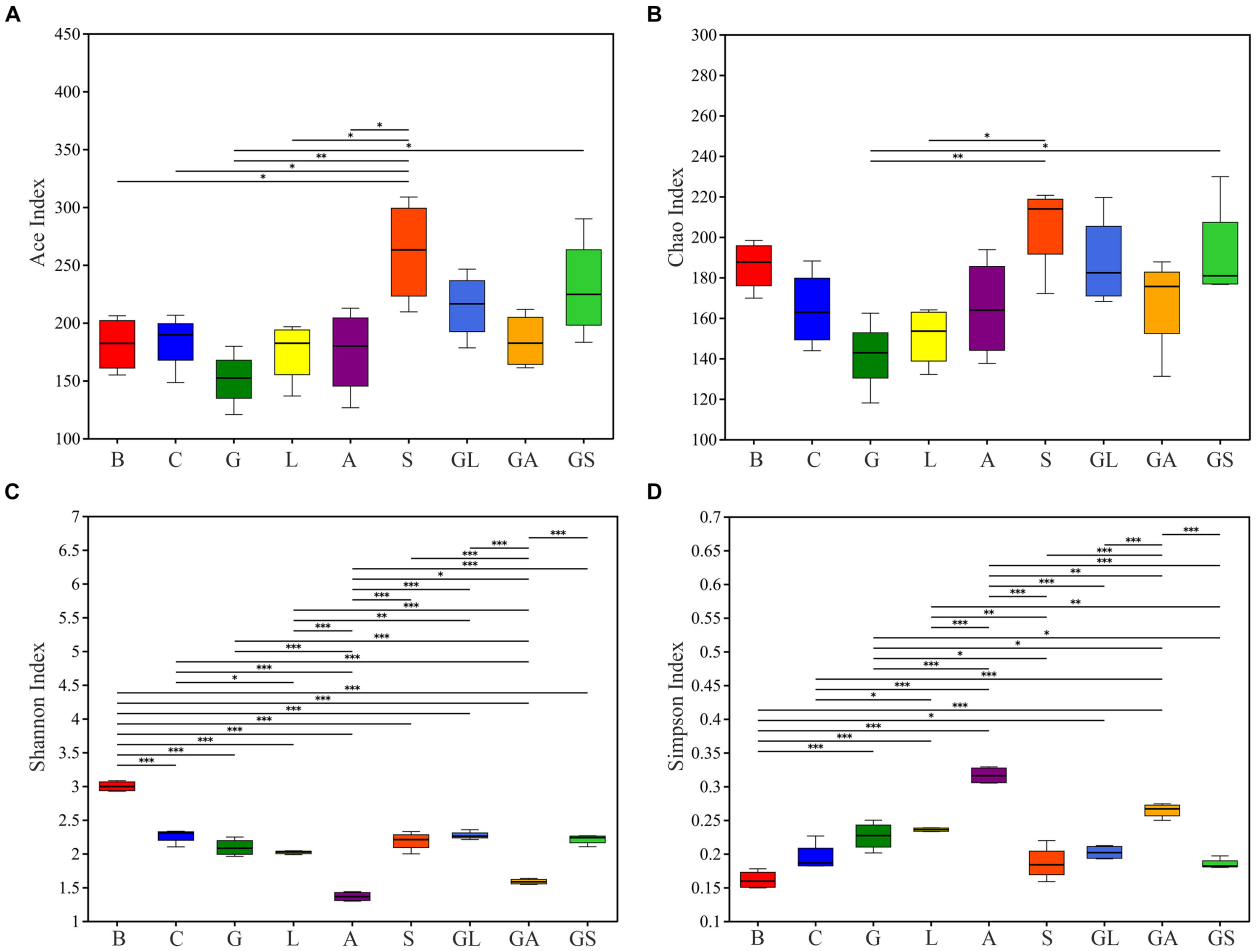
Differences in bacterial community diversity and richness between fresh mulberry and mulberry silage. **(A)** Ace index. **(B)** Chao index. **(C)** Shannon index. **(D)** Simpson index. C, control; G, glucose; L, lactic acid bacteria; A, formic acid; S, salts; GL, glucose + lactic acid bacteria; GA, glucose + formic acid; GS, glucose + salts.

In total, these analyses entailed the evaluation of 36 samples (4 fresh mulberry, 32 silages), yielding 1,316,664 high-quality reads with an average of 36,574 reads per sample. These reads were clustered into 397 OTUs, using the criterion of 97% similarity. The Good’s coverage across these samples was ~0.99 in all cases (Supplementary Table S1). Alpha diversity analyses are presented in Figure 1, revealing similar trends in Ace and Chao1 indices across samples, with these values being highest in the S group, followed by the GS and GL groups, while they were lowest in the G group. Shannon index values declined in all treatment groups following ensiling, whereas Simpson index values rose. Among the silage treatment groups, lower Shannon indices and higher Simpson indices were observed in the A and GA groups.

**Figure 2:**
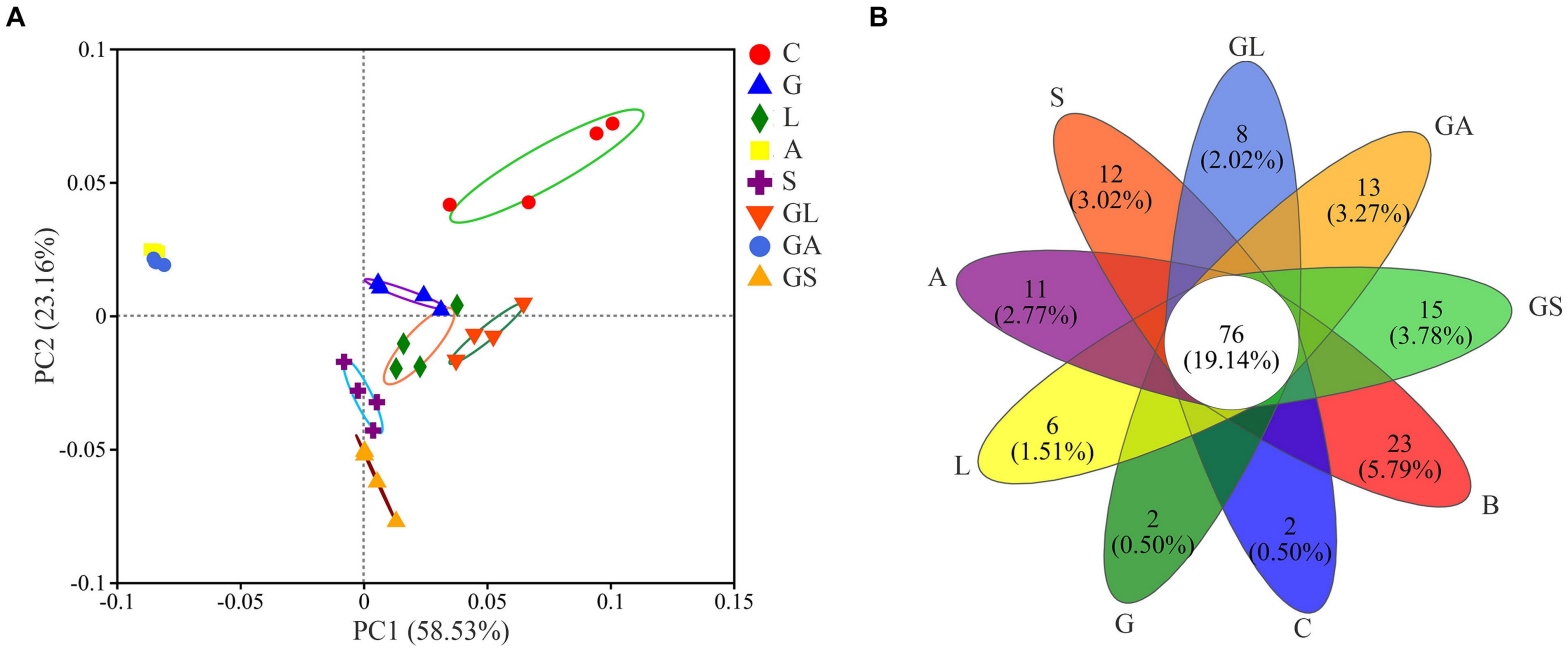
**(A)** Principle coordinate analysis (PCoA) of the bacterial community in silage samples based on Weighted Unifrac distance. **(B)** Venn diagram depicting unique or common bacterial OTUs in silage samples. C, control; G, glucose; L, lactic acid bacteria; A, formic acid; S, salts; GL, glucose + lactic acid bacteria; GA, glucose + formic acid; GS, glucose + salts.

PCoA analyses revealed clear separation among samples from different groups (Figure 2A). PC1 and PC2 were, respectively, found to explain 58.53 and 23.16% of the total change. Groups A and GA were separated from the C group and the five other groups by the greatest distance, whereas the distance among these remaining five groups was relatively similar. In total, 76 OTUs were shared across all treatment groups, while the B, C, G, L, A, S, GL, GA, and GS groups each exhibited 22, 2, 2, 6, 11, 11, 8, 13 and 15 unique OTUs, respectively (Figure 2B).

**Figure 3:**
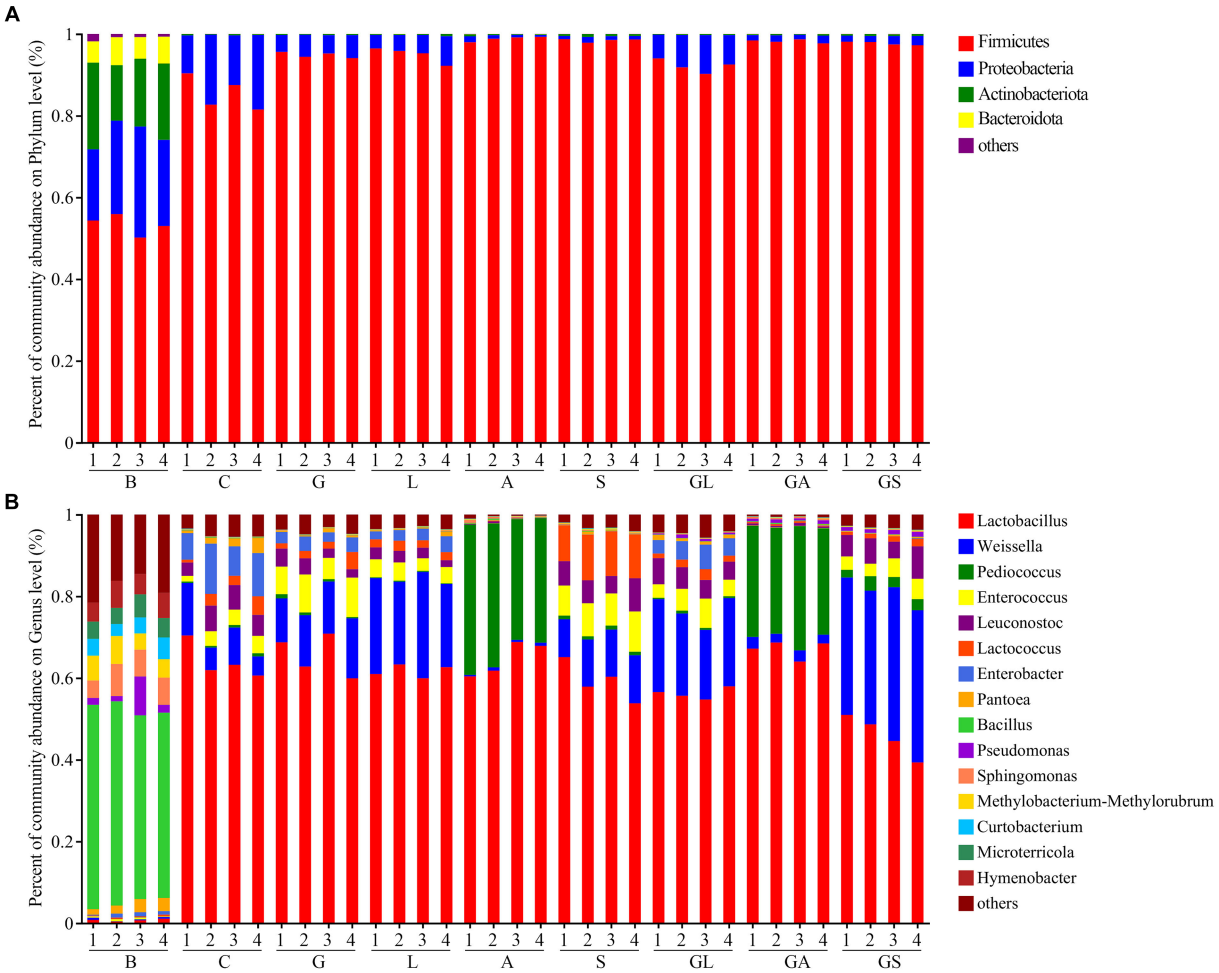
Relative abundance of bacterial composition in fresh mulberry and mulberry silage at the **(A)** phylum and **(B)** genus level. C, control; G, glucose; L, lactic acid bacteria; A, formic acid; S, salts; GL, glucose + lactic acid bacteria; GA, glucose + formic acid; GS, glucose + salts.

The relative phylum- and genus-level bacterial abundance in the analyzed samples is illustrated in Figure 3. The Firmicutes and Proteobacteria phyla were dominant in the fresh samples (53.34 and 22.15%, respectively), while in silage samples the relative abundance of these phyla had shifted to 95.07 and 4.58%, respectively (Supplementary Table S2). In fresh samples, the domainant genera were *Bacillus* (47.56%), *Sphingomonas* (6.34%) and *Hymenobacter* (5.60%) (Supplementary Table S3), while *Lactobacillus* (range: 45.86 to 67.08%) dominated in silage samples. The second most abundant genera in samples from the C group was *Enterobacter* (9.13%), while in samples from the G, L, S, GL, and GS groups it was *Weissella* with a relative abundance of 12.70, 22.53, 11.04, 20.39, and 35.31%, respectively. The second most abundant genus in the A and GA treatment groups was *Pediococcus,* with respective relative abundances of 33.02 and 27.41%.

**Figure 4:**
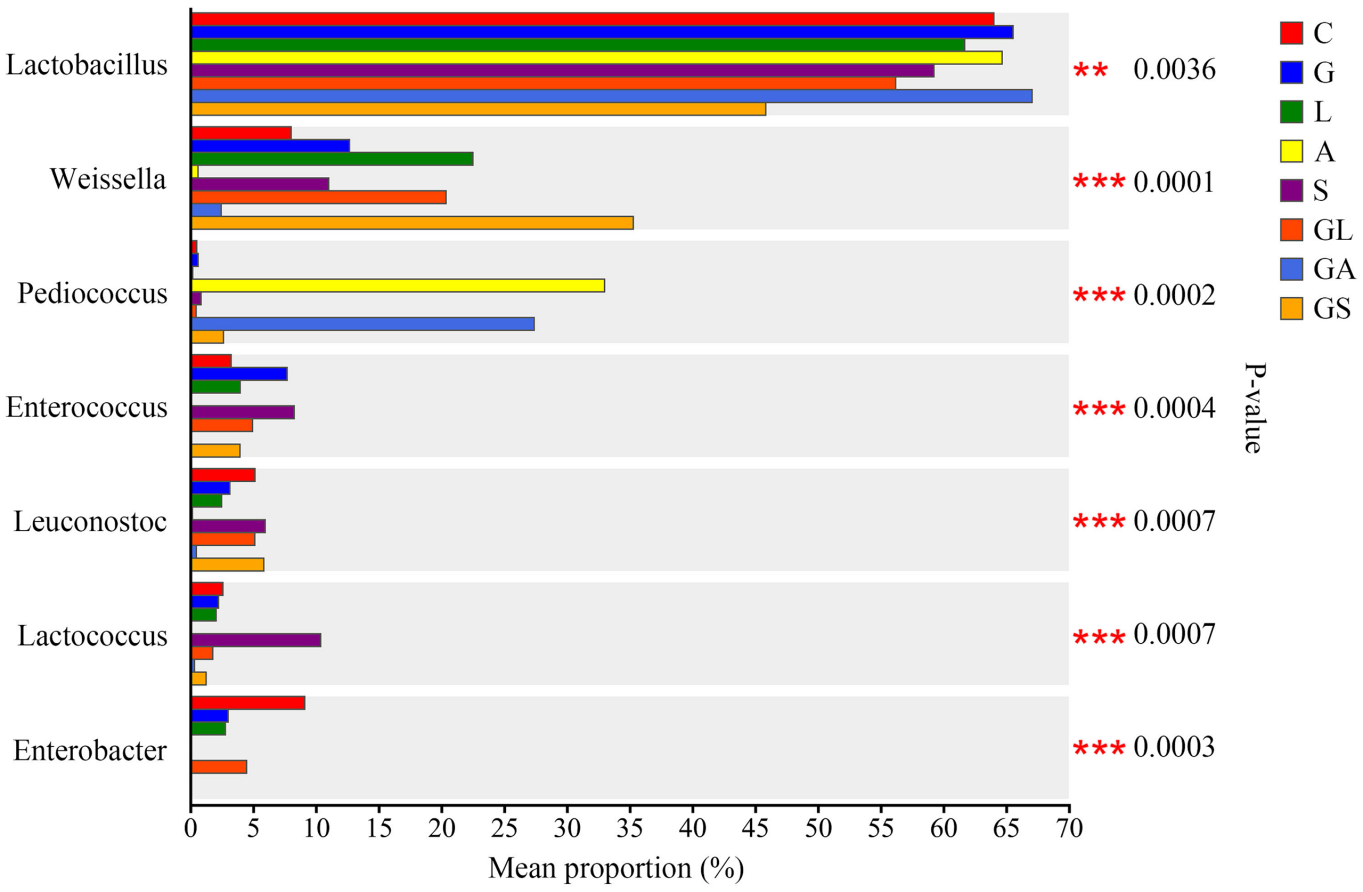
Comparison of different bacteria among the eight treatments. C, control; G, glucose; L, lactic acid bacteria; A, formic acid; S, salts; GL, glucose + lactic acid bacteria; GA, glucose + formic acid; GS, glucose + salts. ^∗^*p* < 0.05; ^∗∗^*p* < 0.01.

Genus-level comparisons of the different bacteria in this study are presented in Figure 4. The levels of *Lactobacillus* were lower in the GS group compared with the remaining seven groups (*p* < 0.01), which were fairly similar to one another. Increased proportions of *Weissella* were observed in the L, GL, and GS groups relative to the C group (*p* < 0.01). The A and GA groups exhibited increased *Pediococcus* abundance together with decreased abundance of *Weissella*, *Enterococcus, Leuconostoc*, *Lactococcus*, and *Enterobacter* relative to the remaining six groups (*p* < 0.01). The proportion of *Lactococcus* in the S group was elevated relative to that in the other groups (*p* < 0.01), while the proportion of *Enterobacter* in the C group was elevated in comparison with the other groups (*p* < 0.01).

### 3.5 Correlation analyses

**Figure 5:**
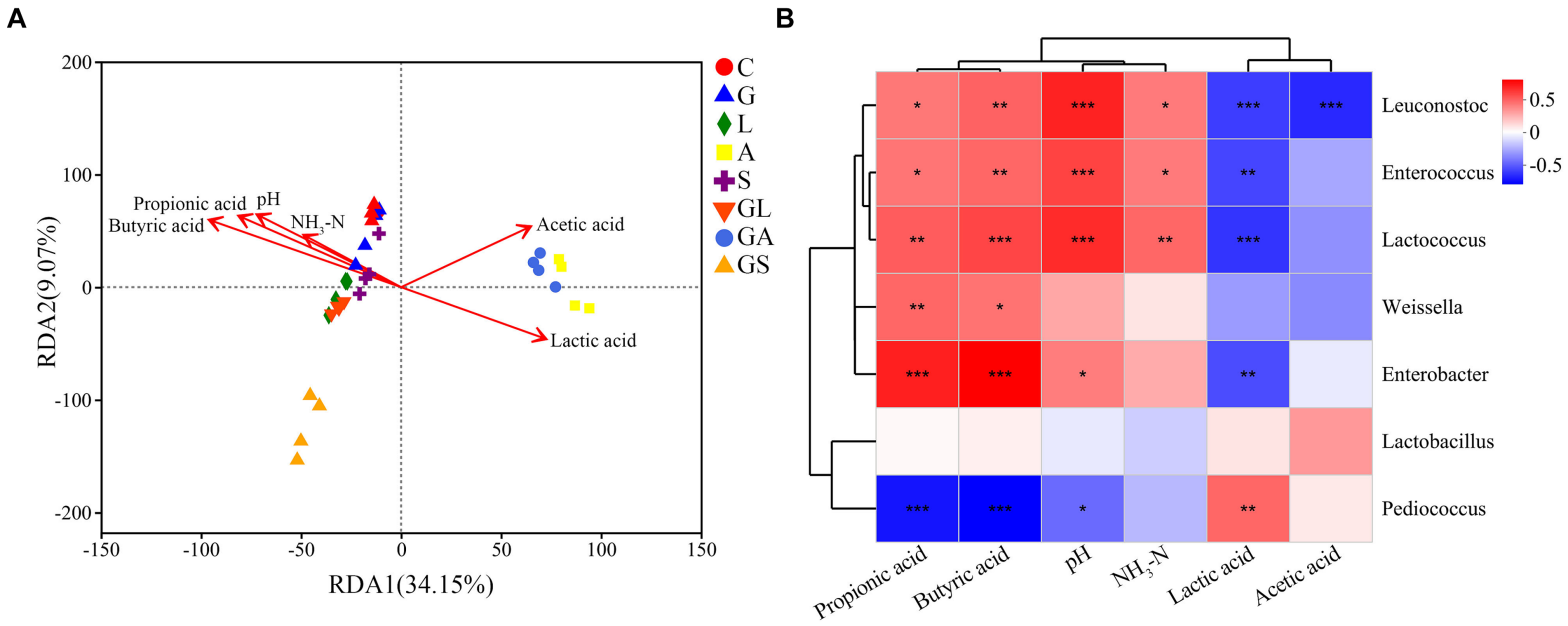
**(A)** Redundancy analysis (RDA) of bacterial data (symbols) and fermentation characteristics (arrows). **(B)** Correlation matrix between fermentation characteristics and microbial composition at the genus level. Positive correlations are shown in red, and negative correlations are shown in blue. Intensity of color is proportional to Pearson correlation coefficient. C, control; G, glucose; L, lactic acid bacteria; A, formic acid; S, salts; GL, glucose + lactic acid bacteria; GA, glucose + formic acid; GS, glucose + salts. ^∗^*p* < 0.05; ^∗∗^*p* < 0.01; ^∗∗∗^*p* < 0.001.

RDA analyses indicated that pH, NH_3_-N, and levels of lactic, acetic, and propionic acids markedly affected bacterial community structures (*p* < 0.05, Figure 5A). Samples in groups A and GA were positively associated with both lactic and acetic acids, while most of the other samples showed a negative association with these acids.

Spearman’s rank correlation analyses revealed positive correlations between *Leuconostoc*, *Enterococcus*, and *Lactococcus* and pH, NH_3_-N, propionic acid, and butyric acid levels (*p* < 0.05, Figure 5B) while the bacteria showed negative relationships with lactic acid levels (*p* < 0.01). *Weissella* was positively associated with propionic acid and butyric acid levels (*p* < 0.05), while a positive correlation was seen between *Enterobacter* and pH, propionic acid, and butyric acid levels (*p* < 0.05) and a negative association with lactic acid levels (*p* < 0.05), while *Pediococcus* exhibited the opposite trend.

## 4 Discussion

Greater CP and lower cell wall content (NDF, ADF, and ADL) can often serve as indicators associated with high-quality forage (Huang et al., 2019). Fresh whole-plant mulberry exhibited a CP content of 14.48% in this study, in line with values reported for traditional forage such as silage derived from alfalfa and soybean (Ni et al., 2018; Özüretmen et al., 2022). The higher CP contents, together with the low ADF and NDF content observed in whole-plant mulberry, suggests that it may be a valuable protein-rich forage option for ruminants. However, whole-plant mulberry exhibited lower CP content than that for mulberry leaves (Wang et al., 2019a; He et al., 2020), together with higher NDF and ADF content, potentially suggesting that the nutritional composition of mulberry plants varies across various parts of these plants. Relative to the fresh material, mulberry silages exhibited expected reductions in DM and WSC content. This is attributable to oxygen consumption by plant cells and aerobic microorganisms during the early stages of ensiling together with the fermentation of WSC into lactic acid by LAB over the course of ensiling (Dunière et al., 2013). The observed decreases in silage NDF, ADF, and ADL content were likely primarily attributable to the hydrolytic breakdown of cell walls by organic acids and enzymes produced during ensiling (Larsen et al., 2017).

Ensiling entails inevitable changes in chemical composition resulting from soluble carbohydrate conversion into organic acids and the breakdown of proteins and fibers (Wei et al., 2021). The higher levels of DM content in the A, S, GA, and GS groups suggest lower DM loss primarily stemming from the ability of formic acid and salts (sodium benzoate + potassium sorbate) to more efficiently inhibit the growth of undesirable microbes, thereby minimizing nutrient loss (Muck et al., 2018). As reported previously (Li et al., 2022; Wu C et al., 2022; Zhao et al., 2022), additives failed to impact NDF content in this study. Cheng et al. (2022), however, found that the *L. plantarum* inoculation of paper mulberry silage led to an increase in NDF content after ensiling for 60 days, while Jiang et al. (2020) reported no change in NDF content in corn silage after treatment with LAB and organic acid after 45 days but a decrease following 90 days of ensiling. Adding formic acid, molasses, and fibrolytic enzymes also reportedly reduced NDF content in Napier grass samples (Desta et al., 2016). These discrepant results may be attributable to differences in the particular grass species, additives, and ensilage times in individual studies. WSC content was increased in groups treated with glucose relative to other groups in the present study, as expected. However, WSC content changes dynamically over the course of ensiling, with the acid hydrolysis of the fiber fraction leading to the release of WSC that can be used by LAB for the production of organic acids (Jiang et al., 2020).

Analyzing pH, organic acid content, and NH_3_-N content can provide valuable insights into silage fermentation quality (Li et al., 2019; Gao et al., 2022). Relative to the C group, the groups treated with additives in the present study exhibited lower pH values and NH_3_-N content, together with higher lactic acid content, suggesting that utilizing these additives led to significant improvements in silage fermentation quality. While the lactic acid content in silage found in the current investigation was consistent with that reported for corn silage in past reports (Liu et al., 2021; Wang et al., 2022), the pH (4.28) was substantially higher than in these prior reports (3.50–3.88). This further emphasizes the challenges of directly ensiling whole mulberry plants, potentially owing to their high buffering capacity, inhibiting drops in the pH of the resultant silage (Hao et al., 2021). Epiphytic LAB counts >10^5^ CFU/g of FM and a WSC content >6% DM are essential for successful fermentation (Oliveira et al., 2017). Adding glucose and LAB can thus overcome the limitations in WSC and epiphytic LAB found in the fresh mulberry material, leading to superior fermentation. Here, the A and GA groups exhibited the best fermentation quality, potentially because the formic acid was able to rapidly lower the pH, thereby inhibiting undesirable microbe growth while providing an environment suitable for the growth of LAB (Lv et al., 2020).

Microbes are vital in the ensiling process such that efforts to monitor the bacterial community during ensiling can inform efforts to better understand and enhance the process of fermentation (Xu et al., 2017). Here, ensiling led to increases in Shannon index values together with decreased Simpson index values, consistent with reduced diversity in bacterial communities and in line with earlier findings (Guan et al., 2018; Wu B et al., 2022). Méndez-García et al. (2015) posited that this may reduction may be linked to the reduction in the pH of the resultant silage, limiting the ability of microbes to grow under acidic conditions. Reductions in unique bacterial OTUs following ensiling were also evident in the present study, consistent with past reports (Ren et al., 2019; Su et al., 2021). The magnitude of this reduction in community diversity was greatest in the A and GA groups, supporting the ability of formic acid to improve the quality of the resultant fermentation more effectively than other additives.

The epiphytic bacterial communities found in the fresh material are closely associated with the type of plant (Jiang et al., 2020). Fresh native grasses, for example, are frequently dominated by *Pantoea*, *Pseudomonas,* and *Erwinia* (Li et al., 2022), whereas *Moringa oleifera* leaves are dominated by *Exiguobacterium*, *Acinetobacter,* and *Pseudomonas* (Wang et al., 2018). In the present study, the dominant genera associated with fresh material were *Bacillus*, *Sphingomonas*, and *Hymenobacter*. *Bacillus* species are capable of producing antifungal compounds and facilitating the degradation of anti-nutritional factors and macromolecular nutrients through exoenzyme secretion (Chi and Cho, 2016). Phyllosphere microbiomes including those associated with fruits and flowers are often rich in *Sphingomonas* and *Hymenobacter* (Olimi et al., 2022). Epiphytic bacteria also exhibit a high degree of sensitivity to climatic, regional, and environmental factors. Guan et al. (2018), for instance, found that epiphytic bacterial communities were significantly correlated with temperature, humidity, and rainfall. Interestingly, despite the variations in epiphytic bacteria reported in the above studies, all of these species decreased sharply in abundance during the ensiling process together with prominent *Lactobacillus* outgrowth.

Additives can have a profound impact on silage quality through their effects on microbial communities present therein. LAB are the primary microorganisms that shape the process of fermentation, and these species can be morphologically classified into bacilli including *Lactobacillus* species, as well as cocci such as *Enterococcus*, *Lactococcus*, *Leuconostoc*, *Pediococcus,* and *Weissella* species (Yang et al., 2022). As expected, greater *Lactobacillus* abundance was evident in the G, A, and GA groups in the present study, likely owing to the addition of glucose as a source of WSC and the ability of formic acid to suppress harmful microbe growth, thereby generating conditions favorable to LAB growth. Higher *Lactobacillus* abundance has also been reported in native grass silage after treatment with molasses (Li et al., 2022) or in corn silage to which formic, acetic, and propionic acids were added at a 7:1:2 ratio (Jiang et al., 2020). Strikingly, the addition of LAB in the L and GL groups led to a decrease in *Lactobacillus* levels relative to the C group while increasing the proportion of *Weissella* in the resultant silage. This coincided with enhanced fermentation quality in these L and GL groups, as determined by the observed reduction in pH and elevation of lactic acid levels. In line with these results, Mu et al. (2021) also determined that adding *Lactobacillus plantarum* and molasses to mixed rice straw and amaranth silage led to a reduction in relative *Lactobacillus* abundance together with an increase in *Weissella* abundance. The mechanisms underlying this observation remain uncertain and warrant further study. The proportions of *Lactobacillus* and *Weissella* in the S and GS groups tended to exhibit similar trends in their variability to those observed in the L and GL groups. This is likely attributable to the ability of potassium sorbate and sodium benzoate to inhibit undesirable microbe growth while also limiting *Lactobacillus* growth, ultimately establishing conditions conducive to *Weissella* growth. Strikingly, all of the additives employed in this study resulted in decreased *Enterobacter* abundance, which is important given that these undesirable bacteria can induce fermentation of lactic acid to produce succinic acid, acetic acid, and certain endotoxins, thereby compromising the nutritional quality of the silage and leading to its contamination (Wang et al., 2022). Relative to other tested additives, the formic acid (A and GA) and salt (S and GS) groups exhibited more effective inhibition of harmful bacterial growth. Adding formic acid also increases the relative abundance of *Pediococcus*, which is frequently utilized to enhance silage quality due to its ability to produce lactic acid and to tolerate acidic conditions (Wang et al., 2019b). Relative to other additives, formic acid thus provides the most effective approach to enhancing *Lactobacillus* and *Pediococcus* abundance while limiting the growth of potentially harmful microbial species.

Using an RDA approach, the relationships between bacterial community composition and fermentation parameters were explored in greater detail. Across all groups, longer arrows were observed for lactic acid and acetic acid, indicating that these two organic acids had the greatest impact on the bacterial communities present in silage, in line with what has been reported in paper mulberry silage (Wu C et al., 2022) and alfalfa silage (Bai et al., 2020). Lactic acid and acetic acid were also found to be positively associated with the samples in groups A and GA, suggesting that adding formic acid led to more effective increases in the content of these organic acids. In line with expectations, lactic acid concentrations were negatively correlated with pH, butyric acid levels, and NH_3_-N concentrations, as lactic acid can reduce pH values and suppress the growth of spoilage-related bacterial species including *Clostridia*, thereby preventing protein degradation and the generation of butyric acid (Wang et al., 2019). Lactate-producing bacteria tended to be positively linked to the concentrations of lactic acid in these samples. However, *Lactobacillus* abundance was not significantly correlated with lactic acid concentrations in the present study, while a negative relationship was reported in alfalfa silage by Wang B et al. (2020). The contributions of *Lactobacillus* to increases in lactic acid levels may be less significant than the contributions of other silage bacteria. *Leuconostoc*, *Enterococcus*, and *Lactococcus* were also found to be negatively associated with concentrations of lactic acid while they were positively associated with pH, butyric acid, and NH_3_-N contents, in contrast to the overall findings. This may suggest that the growth of these beneficial species of bacteria coincided with the increased growth of certain undesirable species. Li et al. (2020) determined that when initial lactic acid fermentation-induced acidification failed to prevent *Clostridia* growth in silage, *Clostridia* fermentation can occur, leading to the production of butyric acid or to ammonia accumulation. Formic acid, in contrast, exhibits a greater capacity for acidification such that it can more effectively suppress the outgrowth of *Clostridia* and certain other undesirable species of bacteria (Lv et al., 2020). Consistent with this possibility, the pH and community diversity indices in the A and GA groups were lower. Additionally, *Pediococcus*, which was more abundant in samples from the A and GA groups, was positively associated with lactic acid concentrations but negatively associated with pH, butyric acid, and NH_3_-N levels, in line with what has previously been described in corn silage by Guan et al. (2018).

## 5 Conclusion

In summary, the present results revealed an increase in DM content in the A, S, GA, and GS treatment groups. Decreased pH and NH_3_-N levels were observed in all additive treatment groups. The A and GA groups presented with the lowest pH values and the greatest lactic acid contents. The various tested additives modified bacterial community structures through increases in the abundance of beneficial bacteria including *Weissella* and *Pediococcus*. A and GA addition was also sufficient to profoundly inhibit *Enterobacter* growth. As such, the additives utilized in this study offer promise as tools to improve whole-plant mulberry silage to varying degrees. A and GA treatments were found to be the most efficient approach to reducing pH levels, increasing lactic acid content, and preventing the growth of undesirable bacteria in ensiled mulberry samples.

## Data availability statement

The 16S rRNA sequence data were submitted to the NCBI Sequence Read Archive (SRA; https://submit.ncbi.nlm.nih.gov/subs/sra/) database with the accession number of PRJNA1024408 for open access.

## Author contributions

LH: Writing – review & editing, Writing – original draft, Conceptualization. FJ: Writing – review & editing, Writing – original draft, Conceptualization. YW: Writing – review & editing, Project administration. HW: Writing – review & editing, Resources, Data curation. HH: Writing – review & editing, Project administration, Data curation. WY: Writing – review & editing, Formal analysis, Data curation. XH: Writing – review & editing, Formal analysis, Data curation. HC: Writing – review & editing, Formal analysis, Data curation. CW: Writing – review & editing, Writing – original draft, Investigation, Funding acquisition. ES: Writing – review & editing, Writing – original draft, Investigation, Funding acquisition.
